# Inhibition of p110δ PI3K prevents inflammatory response and restenosis after artery injury

**DOI:** 10.1042/BSR20171112

**Published:** 2017-09-28

**Authors:** Antonio Bilancio, Barbara Rinaldi, Maria Antonietta Oliviero, Maria Donniacuo, Maria Gaia Monti, Amedeo Boscaino, Irene Marino, Lori Friedman, Francesco Rossi, Bart Vanhaesebroeck, Antimo Migliaccio

**Affiliations:** 1 Department of Biochemistry, Biophysics and General Pathology, University of Campania “L. Vanvitelli”, Naples, Italy; 2 Department of Experimental Medicine, Section of Pharmacology “L. Donatelli”, University of Campania “L. Vanvitelli”, Naples, Italy; 3 Department of Medical Translational Science, University of Naples “Federico II”, Naples, Italy; 4 Department of Histopathology, AORN “Cardarelli”, Naples, Italy; 5 Translational Oncology, Genentech Inc, South San Francisco, CA, U.S.A.; 6 Department of Experimental Medicine, Section of Pharmacology “L. Donatelli”, Regional Centre for Pharmacovigilance and Pharmaco-epidemiology – University of Campania “L. Vanvitelli”, Naples, Italy; 7 Cell Signalling, UCL Cancer Institute, Paul O’Gorman Building, University College London, 72 Huntley Street, London WC1E 6BT, U.K.

**Keywords:** carotid, inflammatory cell, Phosphoinositide 3-kinase (PI3K), p110δ, restenosis

## Abstract

Inflammatory cells play key roles in restenosis upon vascular surgical procedures such as bypass grafts, angioplasty and stent deployment but the molecular mechanisms by which these cells affect restenosis remain unclear. The p110δ isoform of phosphoinositide 3-kinase (PI3K) is mainly expressed in white blood cells. Here, we have investigated whether p110δ PI3K is involved in the pathogenesis of restenosis in a mouse model of carotid injury, which mimics the damage following arterial grafts. We used mice in which p110δ kinase activity has been disabled by a knockin (KI) point mutation in its ATP-binding site (p110δ^D910A/D910A^ PI3K mice). Wild-type (WT) and p110δ^D910A/D910A^ mice were subjected to longitudinal carotid injury. At 14 and 30 days after carotid injury, mice with inactive p110δ showed strongly decreased infiltration of inflammatory cells (including T lymphocytes and macrophages) and vascular smooth muscle cells (VSMCs), compared with WT mice. Likewise, PI-3065, a p110δ-selective PI3K inhibitor, almost completely prevented restenosis after artery injury. Our data showed that p110δ PI3K plays a main role in promoting neointimal thickening and inflammatory processes during vascular stenosis, with its inhibition providing significant reduction in restenosis following carotid injury. p110δ-selective inhibitors, recently approved for the treatment of human B-cell malignancies, therefore, present a new therapeutic opportunity to prevent the restenosis upon artery injury.

## Introduction

Restenosis is the pathophysiological process leading to the failure of revascularization procedures such as angioplasty and stenting in 10–15% of patients [1–4]. Accumulating evidence has shown that recruitment of inflammatory cells to the site of arterial endothelial injury plays a critical role in the pathogenesis of restenosis [5–8]. Vascular injury causes endothelial denudation and adhesion of activated platelets, which induce recruitment of inflammatory cells such as neutrophils, lymphocytes, monocytes and mast cells, promoting vascular smooth muscle cells (VSMCs) proliferation, migration and neointimal growth [5–12].

Several approaches such as artery stent placement, balloon angioplasty, drug-eluting coronary stents and gene therapy have been attempted to prevent the restenosis upon artery injury [13–17]. However, restenosis after vascular intervention remains an unresolved problem.

Vascular injury activates various cell signalling pathways including phosphoinositide 3-kinase (PI3K) [18–23]. Mammals have eight isoforms of PI3K, which have been divided into three classes [18]. Class I PI3Ks phosphorylate the 3-OH of phosphatidylinositol(4,5)bisphosphate (PIP2) to generate the lipid second messenger phosphatidylinositol(3,4,5)trisphosphate (PIP3). Class I PI3Ks comprise the IA and IB subclasses. The class IA PI3Ks comprise three catalytic subunits (p110α, p110β, p110δ) and five regulatory subunits, collectively known as p85s. The latter bind to p-tyrosine in membrane-bound receptors and/or adaptor proteins, inducing the translocation of the cytosolic p110/p85 complex to the plasma membrane to generate PIP3. This lipid is a docking site for several signalling proteins, most notably Akt/PKB, which regulates multiple biologic processes such as migration, growth and proliferation. The single Class IB catalytic subunit (p110γ) is bound to a p101/p84 regulatory subunit and signals downstream of G-protein-coupled receptors. p110γ is expressed in inflammatory cells and in vascular systems such as endothelial cells and VSMCs, and modulates intimal hyperplasia after vascular injury through Th1 response [19]. p110δ is mainly expressed in leucocytes whereas p110α and p110β are ubiquitously expressed. The use of PI3K isoform specific inhibitors and PI3K gene targeted mice have revealed non-redundant functions of the Class I PI3K isoforms [24] and PI3K isoforms are considered therapeutic targets in different diseases, mainly cancer and inflammation [25 ]; p110δ regulates B- and T-cell functions, mast cells activation and macrophages proliferation and migration [26–28]. A p110δ inhibitor has recently been approved for use in human B-cell malignancies [29].

To address the role of p110δ activity in leucocytes in the pathogenesis of restenosis, we have used gene-targeted mice in which the p110δ PI3K has been inactivated by a knockin (KI) mutation (p110δ^D910A/D910A^) in the ATP-binding site, leading to the production of a catalytically inactive p110δ protein [26] in a model of restenosis. We have also used PI-3065, a recently reported pharmacological inhibitor of p110δ PI3K [30].

Here, we report that inhibition of p110δ PI3K in a mouse model of vascular injury leads to a striking reduction in inflammatory cells recruitment and intimal hyperplasia in injured carotid arteries, resulting in strong reduction in restenosis. Therefore, by targeting the inflammatory responses after artery injury using small molecule inhibitors of p110δ PI3K may be a potential therapeutic strategy to prevent and/or reduce restenosis.

## Materials and methods

### Animals

Homozygous p110δ^D910A/D910A^ mice were generated as described [26] and maintained on the C57/BL6 background. Wild-type (WT) littermates generated from heterozygous p110δ^WT/D910A^ intercrosses were used as controls. All experimental procedures were performed according to the GLP guidelines (Italian Law Decree 116/92 issued by the Italian Ministry of Health, as well as EC laws) and to the guidelines from Directive 2010/63/EU of the European Parliament on the protection of animals used for scientific purposes. Protocols relating to the research described were reviewed and approved by the Animal Care and Use Committee of the University of Campania “L. Vanvitelli”, Naples, Italy. All animals were acclimatized and quarantined for at least 1 week before undergoing surgical injury and were housed in individually ventilated cages under controlled conditions (12–12 h light-dark cycle; room temperature: 20 ± 2°C; humidity: 55–60%) with chow and tap water available *ad libitum*. All efforts were made to minimize animal suffering and to reduce the number of animals used.

### Mouse model of carotid artery injury

Mice were anaesthetized with an intraperitoneal injection of ketamine hydrochloride (100 mg/kg), supplemented with xylazine (2.5 mg/kg) as needed. Vital signs were monitored under anaesthesia and sterile techniques were used throughout the procedure. A median incision of 2-cm length was made in the anterior neck region and the carotid injury performed as previously described in rats [31,32]. In brief, neck muscles were shifted laterally and the trachea was identified. To perform the arterial injury, the left carotid artery was visualized and isolated downstream the bifurcation until 1 cm, which was encircled by two 6.0 silk sutures. In the middle of this carotid area, a plastic scanlom clamp for coronary artery bypass grafting was placed for 5 s on the carotid artery to cause a crushing lesion to the vessel. At the same location, equidistant from the extremities of the carotid, a 0.3-mm longitudinal incision was made into the full thickness of the artery. The incision did not emerge from the other side of the vessel. Haemostasis was obtained with a single adventitial 9.0-gauge polypropylene stick, used as a point of suture and by compression with cotton swabs and 2-mm oxyl-methyl-cellulose. The polypropylene stick was used afterwards as a reference frame for experimental investigations. The blood pulsation of the injured carotid was checked by distally to the incision; the skin was then reapproximated by reabsorbable suture. checked by distally to the incision and the skin was stitched by reabsorbable suture. Antibiotic ticarcillin was administered with an intraperitoneal injection (5 mg/kg) to all the mice at the end of surgical procedure and as analgesic mice were treated with ketoprofen (5–10 mg/kg i.p.) for at least 48 h. At different time points (0, 14 and 30 days; *n*=10 for each time) after vascular injury, the injured and uninjured carotid arteries were removed from all the mice under anaesthesia with ketamine hydrochloride (100 mg/kg) and xylazine (2.5 mg/kg), and processed as described below. In the present study, we have reproduced the model of carotid injury for the first time, previously described in rats [4,31,32], in a mouse model by reducing the diameter of the incision, the time and the width of the carotid injury. The experimental model described herein is a validated arteriotomy model of surgical injury in the mouse that mimics the damage occurring, for example during arterial graft or endarterectomy interventions. This model of carotid injury is an alternative to balloon angioplasty and is more invasive than angioplasty as it affects all the three layers of carotid artery [4].

### *In vivo* PI3K-inhibitor studies

PI-3065 (provided by Genentech) was prepared for *in vivo* assays in 0.5% methylcellulose with 0.2% Tween 80 as described [30]. Twenty-four male WT C57/BL6 mice (20–25 g; Envigo, Italy) were treated by oral gavage with PI-3065 (75 mg/kg, once daily) or vehicle (0.5% methylcellulose with 0.2% Tween 80), from 2 days before until 30 days after carotid injury. Six mice treated with the PI3K inhibitor and six mice treated with vehicle were killed at 0 h after surgical procedure while the other mice (six for each group) treated with the inhibitor or vehicle were killed 30 days after carotid injury. The injured and uninjured carotids from all the mice were fixed in 10% formalin for subsequent histological analysis.

### Histomorphometric analysis and immunohistochemical staining

Carotid arteries were harvested, fixed in 4% formalin and processed for paraffin embedding and Haematoxylin and Eosin (H&E) staining. Serial clusters of carotid arteries were obtained from the distal branch point for morphometry and immunohistochemical analyses. In brief, the location of the sections analysed was identified using the point of suture marked by the 9.0 polypropylene stick. A 2-mm part of the injured carotids up and down from the point of suture was used to perform the histological and immunohistochemical analyses. Sections (ten for each carotid) were stained with Weigert-Van Gieson (W-VG) method (Diapath) for elastic fibres and connective tissue [33]. The extent of the injury was quantified by evaluating the thickening of the neointima: the areas surrounded by the external elastic lamina (EEL area), the internal elastic lamina (IEL area) and by measuring the lumen area. Other areas were calculated as follows:

medial area=EEL area-IEL area

neointimal area=IEL area-lumen area

$$\text{neointima-to-media ratio (I/M)}=\frac{\text{neointimal area}}{\text{medial area}}$$

The circumferential length of the EEL and IEL was also measured to determine negative remodelling. Morphometric analysis was performed using a Nikon Eclipse 1000 microscope and the software NISElements 3 (Nikon Instruments, Tokyo, Japan) [34].

### Immunohistochemistry

Deparaffinized slides were treated with the following monoclonal antibodies: Ventana-prediluted monoclonal anti-CD45 (Ventana), anti-CD68 (Ventana), anti-α-smooth muscle actin (Ventana), anti-CD3 (Roche), anti-CD4 (Roche), anti-CD8 (Dako). Antibody incubations and staining were performed on the automated Ventana BenchMark(R) XT slide stainer. Sections were counterstained with H&E. As negative controls, the same procedures were performed without the primary antibodies. All slides were read independently by two investigators. The percentage of immunopositive cells per total number of cells, for each antibody, was determined under a microscope at 400× magnification, and averaged on three sections for carotid artery.

### Isolation and culture of VSMCs

Mouse VSMCs were isolated as reported [35]. Briefly, in mice under anaesthesia with ketamine hydrochloride (100 mg/kg) and xylazine (2.5 mg/kg) aortae were dissected from six WT mice from their origin at the left ventricle to the iliac bifurcation, perfused with PBS and removed. Aortae were placed in a Petri dish with Fungizone solution and the adventitia was removed using a dissection microscope. Aortae were cut into pieces and then digested with 0.3% collagenase solution in a tissue-culture incubator for 5 h. DMEM culture medium with 10% FBS was added to stop collagenase digestion. The cells were washed twice with the culture medium and seeded in culture dishes coated with 10 μg/ml human fibronectin (BD). Primary cultures were trypsinized (0.1% trypsin, Lonza) when confluent, followed by incubation of cells in DMEM with 10% FBS at 37°C in 5% CO_2_ for 5–7 days.

### Measurement of VSMCs proliferation

Primary mouse VSMCs (2.5 × 10^4^) were seeded in 96-well plates coated with 10 μg/ml human fibronectin (BD). Primary VSMCs were washed twice with PBS and placed in serum-free culture medium or 10% FBS in the absence or in presence of increased concentrations of IC87114 (Merck Serono, Geneva, CH), followed by measurement of proliferation assay using the WST-1 cell proliferation kit (Roche) according to the manufacturer’s instructions. Stocks of IC87114 for *in vitro* assays were prepared in DMSO.

### Statistical analysis

All data are presented as mean ± S.E.M. unless otherwise stated. The Mann–Whitney two-tailed U test was used to calculate the statistical significance of animal experiments. Results were considered significant at a value of *P*<0.05. All authors have read and agreed to the manuscript as written.

## Results

### Effect of p110δ PI3K inactivation on neontimal hyperplasia in injured carotid arteries

**Figure 1:**
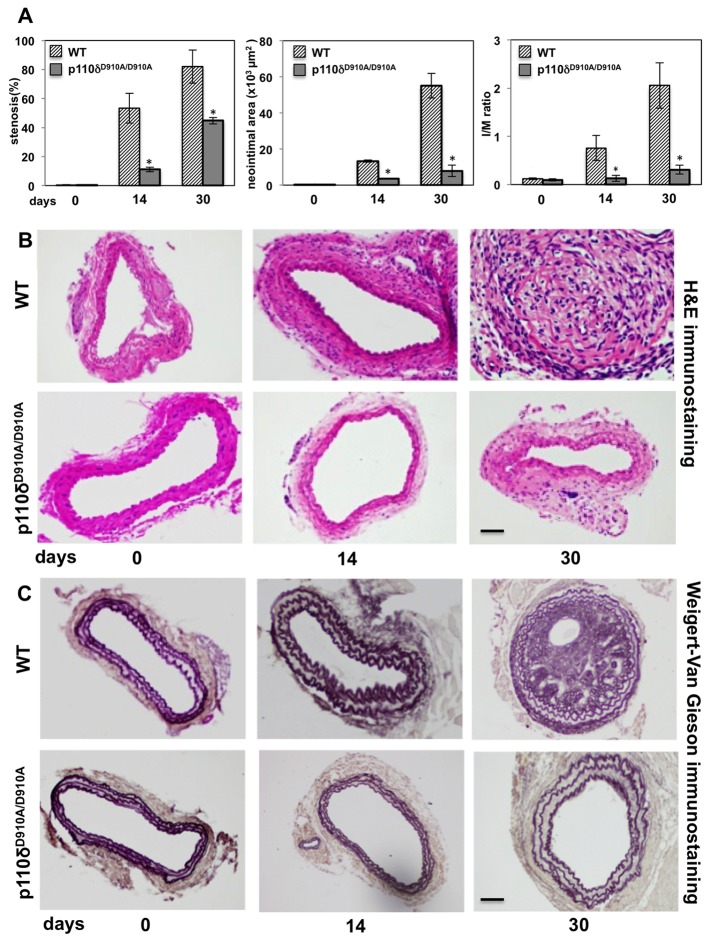
Effect of p110δ PI3K inactivation in injured carotid arteries after surgery (**A**) Left panel: percentage of arterial stenosis; middle panel: quantificative morphometric analysis of neointimal area expressed in square pixels; right panel: I/M ratio index in control (WT) and p110δ^D910A/D910A^ mice. Data are expressed as mean ± S.E.M. (*n*=10 per genotype); *P<0.01 compared with WT. (**B**) Representative H&E staining of cross-sections from injured carotid arteries of WT and p110δ^D910A/D910A^ mice, 0, 14 and 30 days after injury (*n*=10 per genotype). Scale bar =50 μm. (**C**) Representative W-VG staining of cross-sections from injured carotid arteries of WT and p110δ^D910A/D910A^ mice, 0, 14 and 30 days after injury. Scale bar =50 μm.

Given that neointimal appearance is a hallmark of endothelial damage, we first investigated the impact of p110δ inactivation on neointimal formation using an established *in vivo* carotid injured model [31], analysing tissues at 0, 14 or 30 days after injury in carotid arteries of WT and p110δ^D910A/D910A^ mice. This was accomplished by morphometric analysis of cross-sections of carotid artery lesions by H&E staining to assess the whole carotid thickness and lumen/wall ratio (Figure 1A,B) and by W-VG staining to analyse specifically the elastic lamina (Figure 1C).

Morphometric analysis revealed a clear time-dependent increase in lumen stenosis in injured carotid arteries in WT mice, compared with a much reduced lumen stenosis in p110δ^D910A/D910A^ mice. For example, WT mice showed a 53% stenosis 14 days after injury, compared with 11% in injured carotid arteries from p110δ^D910A/D910A^ mice (Figure 1A, left panel). Thirty days after injury, the lumen stenosis increased up to 82% in WT mice compared with 45% in p110δ^D910A/D910A^ mice (Figure 1A, left panel). Representative images of injured H&E-stained arteries from WT and p110δ^D910A/D910A^ mice, 0, 14 and 30 days after injury, are shown in Figure 1B.

Morphometric analysis by W-VG staining revealed that in WT mice, the neointimal lesion completely occluded the vessel lumen 30 days after carotid injury, while this was significantly lower in p110δ^D910A/D910A^ mice (Figure 1C). Neointimal area formation and I/M ratio were also significantly reduced in p110δ^D910A/D910A^ mice compared with WT mice, both at 14 and 30 days after carotid injury (Figure 1A). Specifically, 14 days after carotid injury, the neointimal area was 13.2 × 10^3^ μm^2^ in WT mice compared with 3.5 × 10^3^ μm^2^ in p110δ^D910A/D910A^ mice (Figure 1A, middle panel). The findings were even more impressive 30 days after injury when neointimal area was 55 × 10^3^ μm^2^ in WT compared with 7.8 × 10^3^ μm^2^ in p110δ^D910A/D910A^ mice (Figure 1A, middle panel). The I/M ratio, i.e. the ratio between the thickness of arterial intima compared with the thickness of media layer of carotid artery, is used as an index of the intimal hyperplasia; this value was 0.8 and 0.13 (after 14 days) and 2.1 and 0.3 (at 30 days after carotid injury) in WT and p110δ^D910A/D910A^ mice respectively (Figure 1A, right panel). Representative images of injured carotid arteries at all experimental time points are shown in Figure 1B,C. Importantly, there was no increase in medial area in injured carotid arteries in WT mice compared with p110δ^D910A/D910A^ mice at all time points analysed, with no changes in intima, medial area, I/M ratio and thickness of elastic lamina in uninjured carotids of WT compared with p110δ^D910A/D910A^ (results not shown).

Taken together, these results demonstrate that p110δ PI3K plays a significant role during vascular lesion formation in restenosis.

### p110δ PI3K inactivation reduces VSMCs content of neointimal formation

**Figure 2:**
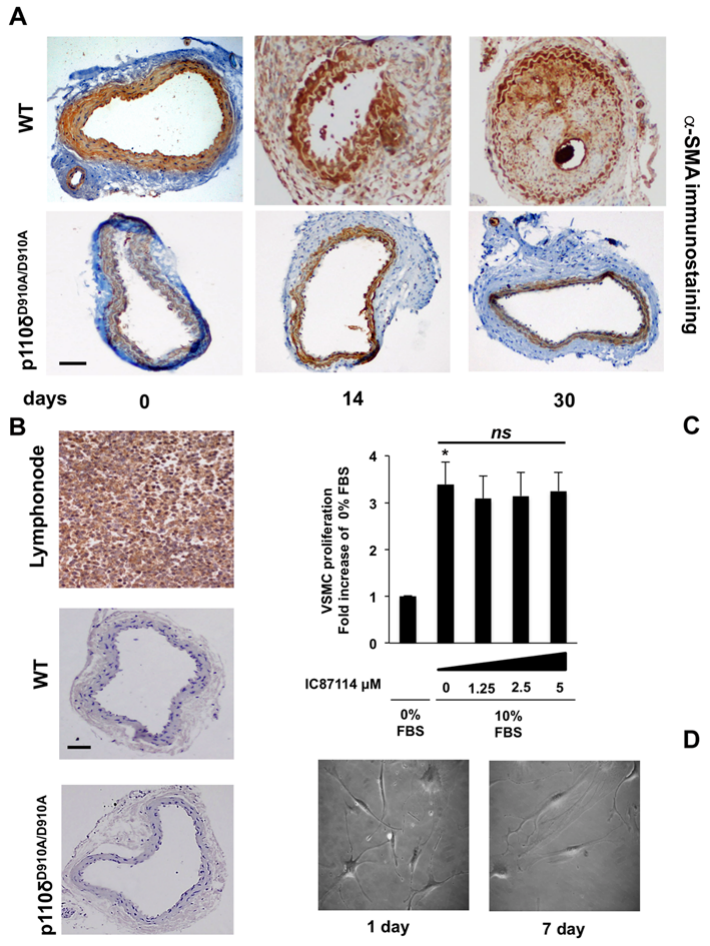
Effect of p110δ PI3K inactivation on VSMCs in neointimal formation (**A**) Representative α-smooth muscle actin (α-SMA) staining of cross-sections from injured carotid arteries of WT and p110δ^D910A/D910A^ mice, 0, 14 and 30 days after injury (*n*=10 per genotype), indicating that the major cellular component in the neointimal area consists of VSMCs. Scale bar =50 μm. (**B**) Representative anti-p110δ staining of cross-sections from lymph nodes and injured carotid arteries in WT and p110δ^D910A/D910A^ mice, 0 days after injury. Scale bar =50 μm. (**C**) Effect of p110δ inhibition on primary VSMCs proliferation. Cells were unstimulated or stimulated with 10% FBS in the presence or absence of the indicated doses of IC87114. Proliferation rate is expressed as fold increase over unstimulated cells. Data are expressed as mean ± S.E.M. of three independent experiments; *P<0.05; ns, non-significant statistical differences. (**D**) Phase-contrast microscopy of representative images of primary VSMCs seeded on Petri dishes treated with fibronectin after 1 or 7 days of culture.

It is well established that VSMCs migration and proliferation are responsible for neointima formation [11,36–38]. We therefore assessed the cellular components of neointimal lesions in WT and in p110δ^D910A/D910A^ mice after carotid artery injury. Immunohistochemical staining with α-smooth muscle actin (α-SMA) of carotid artery cross-sections confirmed that VSMCs were the major cellular component of neointimal formation in carotid arteries in WT mice after injury at 14 and 30 days, whereas the presence of these cells was almost undetectable in sections from p110δ^D910A/D910A^ mice (Figure 2A).

We next investigated whether p110δ was expressed in the different cellular components of the carotid artery wall. Analysis of immunohistochemistry (IHC) staining with an antibody directed against p110δ PI3K reveals that this PI3K is not expressed in the carotid wall, in contrast with its clear presence in lymph nodes (Figure 2B). Moreover, IC87114, a p110δ PI3K-selective inhibitor [28], did not affect VSMCs proliferation (Figure 2C), even at saturating doses of this compound. Furthermore, VSMCs did not show detectable changes in number or shape throughout a 7-day culture (Figure 2D). Taken together, these data show that p110δ PI3K is likely to play a pivotal role in VSMCs migration after vascular injury by a primary action on the immune cell compartment.

### Effect of genetic inactivation of p110δ PI3K on inflammatory cells recruitment and effect of pharmacological inactivation of p110δ PI3K in injured carotid arteries

**Figure 3:**
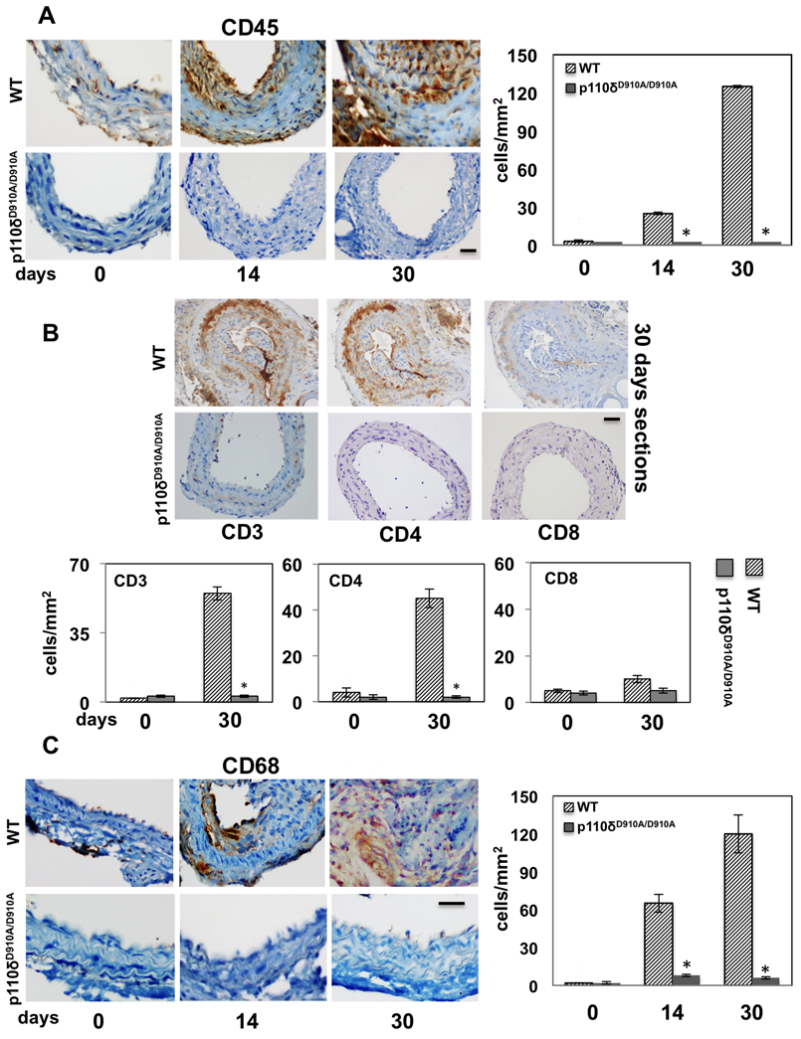
Impact of p110δ PI3K inactivation on lymphocyte and macrophage infiltration into injured carotids (**A**) Representative staining with anti-CD45 (lymphocytes) of cross-sections from injured carotid arteries of WT and p110δ^D910A/D910A^ mice, 0, 14 and 30 days after injury. Scale bar =50 μm. Bar graphs show the number of CD45-positive cells. Data are expressed as mean ± S.E.M. (*n*=10 per genotype); *P<0.05 compared with WT. (**B**) Impact of p110δ PI3K inactivation on T-lymphocyte infiltration of lesions of p110δ^D910A/D910A^ mice, 30 days after carotid artery injury. Representative staining with antibodies to CD3 (T cells), CD4 (T helper) or CD8 (cytotoxic T cells) of cross-sections from injured carotid arteries from WT and p110δ^D910A/D910A^ mice, 30 days after injury. Scale bar =50 μm. Bar graphs show the number of CD3-, CD4-, CD8-positive cells. Data are expressed as mean ± S.E.M. (*n*=5 per genotype); *P<0.05 compared with WT. (**C**) Representative staining with anti-CD68 (macrophages) of cross-sections from injured carotid arteries of WT and p110δ^D910A/D910A^ mice, 0, 14 and 30 days after injury. Scale bar =50 μm. Bar graphs show the number of CD68-positive cells. Data are expressed as mean ± S.E.M. (*n*=10 per genotype); *P<0.05 compared with WT.

Vascular injury involves immune cells recruitment to the site of vascular lesions [5–8]. Since we have previously shown that p110δ PI3K plays a key role in modulating immune cells and inflammatory responses in B and T lymphocytes, macrophages and mast cells [26–28], we therefore investigated the impact of p110δ inactivation on the recruitment of immune cells after carotid artery injury. We performed IHC with anti-CD45 to quantify leucocytes accumulation (Figure 3A) and with anti-CD68 to quantify macrophages (Figure 3C). Uninjured carotid arteries did not reveal CD45 and CD68 staining in WT and p110δ^D910A/D910A^ mice at all time points analysed. Control IgG also showed no signal (results not shown). In WT mice, the number of total leucocytes, identified by anti-CD45 immunostaining (Figure 3A), almost undetectable immediately after injury, rose to 28 and 126 cells/mm^2^, 14 and 30 days after carotid injury respectively, whereas no leucocyte infiltration was observed in p110δ^D910A/D910A^ mice (Figure 3A).

We next characterized the T-cell subpopulations at the site of the carotid injury. Analysis of total T cells by staining for the CD3 marker, T-helper cells by staining for CD4 and cytotoxic T cells by staining for CD8, revealed that T-helper cells represent the majority of T cells present in WT carotid 30 days after injury, whereas no T-cell presence was observed in injured carotid arteries from p110δ^D910A/D910A^ mice (Figure 3B).

The behaviour of macrophages, identified by antibodies to CD68, mirrored that of total leucocytes (Figure 3C), with a dramatic increase in macrophages infiltration at 14 and 30 days after injury in WT mice (64 and 122 cells/mm^2^ respectively), whereas no detectable infiltration was observed in p110δ^D910A/D910A^ mice (Figure 3C).

**Figure 4:**
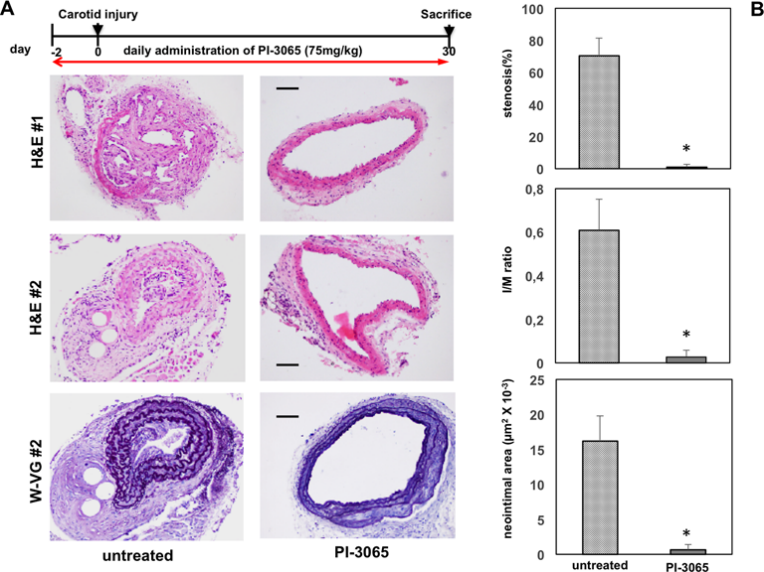
Impact of pharmacological inactivation of p110δ PI3K in injured carotid arteries Top: schematic representation of the procedure used for pharmacological inhibition of p110δ PI3K in WT mice. (**A**) Upper and middle panels: representative H&E staining of cross-sections from two different injured carotid arteries of WT mice, 30 days after injury (*n*=5 per genotype), treated with vehicle (control; left panel) or PI-3065 (right panel). Scale bar =50 μm. Lower panels: representative W-VG staining of cross-sections from injured carotid arteries of WT mice, 30 days after injury (*n*=5 per genotype), treated with vehicle (control; left panel) or PI-3065 (right panel). Scale bar =50 μm. (**B**) Quantificative analysis of stenosis, I/M ratio, neointimal area in indicated mice. Data are expressed as mean ± S.E.M. (*n*=5 per genotype); *P<0.01 compared with untreated.

We next tested the impact of PI-3065, a small molecule, selective inhibitor of p110δ PI3K, on carotid restenosis after injury in WT mice. Daily administration of PI-3065 2 days before the carotid injury until 30 days after induction in the lesions, strongly inhibited the neointimal formation after injury compared with vehicle-treated WT mice (Figure 4).

Taken together, these data support the role for p110δ PI3K in the inflammatory cells recruitment for the pathogenesis of vascular injury. Pharmacological interference with this kinase is possible in the clinic, opening a therapeutic opportunity to interfere with restenosis.

## Discussion

In the present study, we investigated whether p110δ PI3K inactivation protects against restenosis. We provide the first direct *in vivo* evidence, using both genetic and pharmacological approaches, that p110δ kinase activity contributes to immune cells migration to neointimal hyperplasia after carotid injury. p110δ PI3K belongs to a lipid kinase family which regulates multiple biological functions such as proliferation, migration, survival and growth [18,24,39,40]. While p110α and p110β are ubiquitously expressed, p110δ PI3K is mainly expressed in white blood cells [18,24,39]. To address the specific role of p110δ PI3K, p110δ^D910A/D910A^ mice have been generated in which the lipid kinase has been inactivated [26]. p110δ^D910A/D910A^ mice show defects in proliferation and differentiation of B and T lymphocytes [26,28,41]. In addition, p110δ PI3K regulates colony stimulating factor-1-induced migration of macrophages [42] and in LPS signalling in dendritic cells [43]. Recent studies using a p110δ-selective inhibitor in a mouse model of rheumatoid arthritis showed that p110δ plays an anti-inflammatory role in synoviocytes through inhibition of Akt/PKB activation, induced by platelet-derived growth factor and tumour necrosis factor α (TNFα) [44]. Analysis of p110δ^D910A/D910A^ mice in a cerebral stroke model has shown that the p110δ inhibition reduces the production of proinflammatory cytokines such as TNFα, with reduction in the infiltration of inflammatory cells and related neuroinflammatory processes, leading to a considerable improvement in brain functionality [45]. Furthermore, p110δ PI3K regulates Ca^2+^ current and vascular contractility in a mouse model of type I diabetes [22]. p110α PI3K has been recently reported to have a key role in neointima formation after balloon injury of the carotid artery whereas p110β and p110δ PI3K appeared to have no role on VMSCs proliferation and chemotaxis [23]. In addition, p110γ PI3K inhibition has been shown to reduce the vascular restenosis after artery injury with no significant effect on VSMCs proliferation [19]. Consistent with these findings, our results show that p110δ PI3K has a negligible effect on VMSCs proliferation but, on the other hand, has an impressive impact on the inflammatory response, which is likely to precede the arterial lumen narrowing as it is highly enriched in immune cells.

Hence, p110δ PI3K inhibition does not affect VSMCs proliferation in an *in vitro* assay but it is most likely to be involved in immune response, although, it cannot be excluded that p110δ PI3K, even at very low expression, may also contribute to VSMCs proliferation in an *in vivo* context. In the present study, we used a full thickness injury model of carotid wall in p110δ^D910A/D910A^ and WT mice. We observed a significant lumen reduction in the carotid vessels 30 days after carotid injury in almost all the cases analysed in WT mice, while the neointimal thickness was consistently reduced in p110δ^D910A/D910A^ mice at all time points analysed. It is well established that the artery damage induces release of cytokines and growth factors by endothelial cells, VSMCs and blood cells, which are responsible for the chemoattraction of leucocytes, macrophages and dendritic cells and for the proliferation and migration of VSMCs [5–12]. In fact, in injured arteries of WT mice, we observed a dramatically enhanced neointimal formation, mainly composed of VSMCs and a strong inflammatory cellular components but, impressively, these were completely absent from arteries of injured p110δ^D910A/D910A^ mice. In WT mice, the inflammatory cellular components in injured carotid arteries were almost exclusively made up of macrophages and T lymphocytes. Accumulating evidence indicates that the T cells play a key role in the neointimal lesion [19,46–49]. In our model, we found that CD4^+^ T lymphocytes represent the majority of all leucocytes in the neointimal lesion. Nevertheless, we cannot rule out that other cell/tissue components, which express lower level of p110δ PI3K, might contribute to restenosis since previous findings reported that this kinase was also expressed in non-leucocyte cells [50]. In addition, proinflammatory cytokines such as TNFα leads to an increased expression of p110δ PI3K in endothelial cells and in synovial fibroblasts [50]. Independent of where in the organism p110δ is expressed and is functional, the data presented here indicate that p110δ PI3K plays a major role in the inflammatory response to endothelial damage artery. In fact, it is important to keep in mind that systemic pharmacological blockade of p110δ activity is expected to target this kinase everywhere it is expressed in the organism. Prevention of restenosis, essentially due to neointimal formation, is one of the major issues of the treatment of vascular injury caused by different therapeutic manoeuvres such as angioplasty and stent introduction [13]. To control the neointimal formation leading to potential vessel obstruction, metal stents are available coated with immunosuppressive drugs (so-called drug-eluting systems) that have a cytostatic action, including rapamycin derivatives such as sirolimus, everolimus or zotarolimus [14,15]. These drugs are most likely to prevent neointimal hyperplasia by inhibiting inflammatory responses. Alternatively, the use of antimitotic agents like paclitaxel has been proposed to reduce restenosis risk [16], although neointimal formation seems mostly due to monocytes-macrophages infiltration rather than active ‘*in situ*’ proliferation [1]. Among the inflammatory effectors specifically involved in neointimal regeneration, a role of interleukin (IL)-18 [51] and inflammatory mediators such C5a [38] has emerged. Consistent with this finding, an inflammosome adaptor molecule, the apoptosis-associated speck-like protein promotes the development of restenosis and atherosclerosis by increasing IL-18 and IL1-β levels [12]. Here, we show that a selective small molecule inhibitor of p110δ PI3K could be used to prevent restenosis after carotid injury. Indeed, WT mice treated with PI-3065, a p110δ-selective PI3K inhibitor [30], 2 days before the carotid injury until 30 days after carotid injury, greatly reduces restenosis after carotid injury compared with mice treated with vehicle.

Taken together, our data show that inhibition of p110δ PI3K almost completely abolishes the series of events leading to arterial occlusion following carotid damage. Therefore, specific inhibitors of p110δ PI3K, already approved for the human B-cell malignancies and currently under examination in phase III of clinical trials for several pathologies, may have potential for use not only in oncological diseases of white blood cells [29] and immunotherapy of solid tumours [30], but could provide the possibility of using a single-drug approach for the prevention and treatment of stenosis of blood vessels.

However, further work is required to validate the use of p110δ PI3K inhibitors in human disease since recent clinical observations have revealed some unexpected side effects of the p110δ PI3K inhibitor idelalisib, raising a hot debate [52]. It will be of interest to explore if lower doses of p110δ PI3K inhibitors than the currently high doses used in oncology will have anti-inflammatory impact, without the severe side effects reported.

## Abbreviations

Akt: serine/threonine-specific protein kinase
DMEM: Dulbecco's Modified Eagle Medium
EC: European Community
EEL: external elastic lamina
GLP: Good Laboratory Practice
H&E: haematoxylin and eosin
IEL: internal elastic lamina
IHC: immunohistochemistry
IL: interleukin
I/M: neointima-to-media ratio
i.p.: intraperitoneal
LPS: lipopolysaccharide
PIP3: phosphatidylinositol(3,4,5)trisphosphate
PI3K: phosphoinositide 3-kinase
PKB: protein kinase B
Th1: T helper-1
TNFα: tumour necrosis factor α
VSMC: vascular smooth muscle cell
WT: wild-type
W-VG: Weigert-Van Gieson
